# Artificial intelligence fully automated myocardial strain quantification for risk stratification following acute myocardial infarction

**DOI:** 10.1038/s41598-022-16228-w

**Published:** 2022-07-18

**Authors:** Sören J. Backhaus, Haneen Aldehayat, Johannes T. Kowallick, Ruben Evertz, Torben Lange, Shelby Kutty, Boris Bigalke, Matthias Gutberlet, Gerd Hasenfuß, Holger Thiele, Thomas Stiermaier, Ingo Eitel, Andreas Schuster

**Affiliations:** 1 Department of Cardiology and Pneumology, University Medical Centre, Georg-August-University Göttingen, Robert-Koch-Str. 40, 37099 Göttingen, Germany; 2 German Center for Cardiovascular Research (DZHK), Partner Site Göttingen, Göttingen, Germany; 3 University Medical Center Göttingen, Institute for Diagnostic and Interventional Radiology, Georg-August University, Göttingen, Germany; 4 Helen B. Taussig Heart Center, The Johns Hopkins Hospital and School of Medicine, Baltimore, MD USA; 5 Department of Cardiology, Charité Campus Benjamin Franklin, University Medical Center Berlin, Berlin, Germany; 6 Institute of Diagnostic and Interventional Radiology, Heart Center Leipzig at University of Leipzig, Leipzig, Germany; 7 Department of Internal Medicine/Cardiology, Heart Center Leipzig at University of Leipzig, Leipzig, Germany; 8 University Heart Center Lübeck, Medical Clinic II (Cardiology/Angiology/Intensive Care Medicine), University Hospital Schleswig-Holstein, Lübeck, Germany; 9 German Center for Cardiovascular Research (DZHK), Partner Site Hamburg/Kiel/Lübeck, Lübeck, Germany

**Keywords:** Acute coronary syndromes, Myocardial infarction

## Abstract

Feasibility of automated volume-derived cardiac functional evaluation has successfully been demonstrated using cardiovascular magnetic resonance (CMR) imaging. Notwithstanding, strain assessment has proven incremental value for cardiovascular risk stratification. Since introduction of deformation imaging to clinical practice has been complicated by time-consuming post-processing, we sought to investigate automation respectively. CMR data (n = 1095 patients) from two prospectively recruited acute myocardial infarction (AMI) populations with ST-elevation (STEMI) (AIDA STEMI n = 759) and non-STEMI (TATORT-NSTEMI n = 336) were analysed fully automated and manually on conventional cine sequences. LV function assessment included global longitudinal, circumferential, and radial strains (GLS/GCS/GRS). Agreements were assessed between automated and manual strain assessments. The former were assessed for major adverse cardiac event (MACE) prediction within 12 months following AMI. Manually and automated derived GLS showed the best and excellent agreement with an intraclass correlation coefficient (ICC) of 0.81. Agreement was good for GCS and poor for GRS. Amongst automated analyses, GLS (HR 1.12, 95% CI 1.08–1.16, *p* < 0.001) and GCS (HR 1.07, 95% CI 1.05–1.10, *p* < 0.001) best predicted MACE with similar diagnostic accuracy compared to manual analyses; area under the curve (AUC) for GLS (auto 0.691 vs. manual 0.693, *p* = 0.801) and GCS (auto 0.668 vs. manual 0.686, *p* = 0.425). Amongst automated functional analyses, GLS was the only independent predictor of MACE in multivariate analyses (HR 1.10, 95% CI 1.04–1.15, *p* < 0.001). Considering high agreement of automated GLS and equally high accuracy for risk prediction compared to the reference standard of manual analyses, automation may improve efficiency and aid in clinical routine implementation.

Trial registration: ClinicalTrials.gov, NCT00712101 and NCT01612312.

## Introduction

Cardiovascular disease, amongst which acute myocardial infarction (AMI) constitutes a major fraction^1^, has been a leading cause for mortality worldwide during the past decades^2,3^. Therefore, precise risk stratification is a cornerstone in clinical practice to evaluate adequate treatment strategies ranging from drug therapy^4^ to implantable cardioverter-defibrillator (ICD) implantation^5,6^. To date, the treatment decision broadly relies on left ventricular ejection fraction (LVEF) assessment, although data has demonstrated superiority of deformation imaging for risk stratification^7,8^.

Cardiovascular magnetic resonance (CMR) imaging enables precise myocardial deformation assessments including dedicated sequences^9^ as well as post-processing of routinely acquired cine sequences^10^. Although the latter allows reliable deformation imaging without alterations to the CMR protocol and offers incremental value for risk assessment^7^, clinical implementation has been complicated by costly and time-consuming post-processing. Meanwhile, artificial intelligence (AI) based volumetric post-processing has been introduced for automated analyses of CMR cine sequences and demonstrated non-inferiority for major adverse cardiac event (MACE) prediction compared to manual analyses^11^. With the novel availability of AI based deformation imaging, the present project aimed first to assess the reproducibility of automated deformation imaging compared to the reference standard of manual analyses and second to evaluate its value for MACE prediction^7,8,12^ in a large prospectively recruited population of ST-elevation myocardial infarction (STEMI) and non-STEMI patients.

## Materials and methods

### Study population

The patient population of this CMR substudy consisted of patients from two previously published open-label, multicentre trials which included patients referred for CMR imaging following AMI: namely the AIDA STEMI (Abciximab i.v. vs i.c. in ST-elevation Myocardial Infarction, NCT00712101, n = 2065)^13^ and TATORT-NSTEMI (Thrombus Aspiration in Thrombus Containing Culprit Lesions in Non-ST Elevation Myocardial Infarction, NCT01612312, n = 460)^14^ trials. Both studies were approved by the respective ethics committees and the lead ethical institution at the University of Leipzig. The study was conducted according to the principles of the Helsinki Declaration and all research was performed in accordance with relevant guidelines/regulations All patients gave written informed consent before participation.

**Figure 1:**
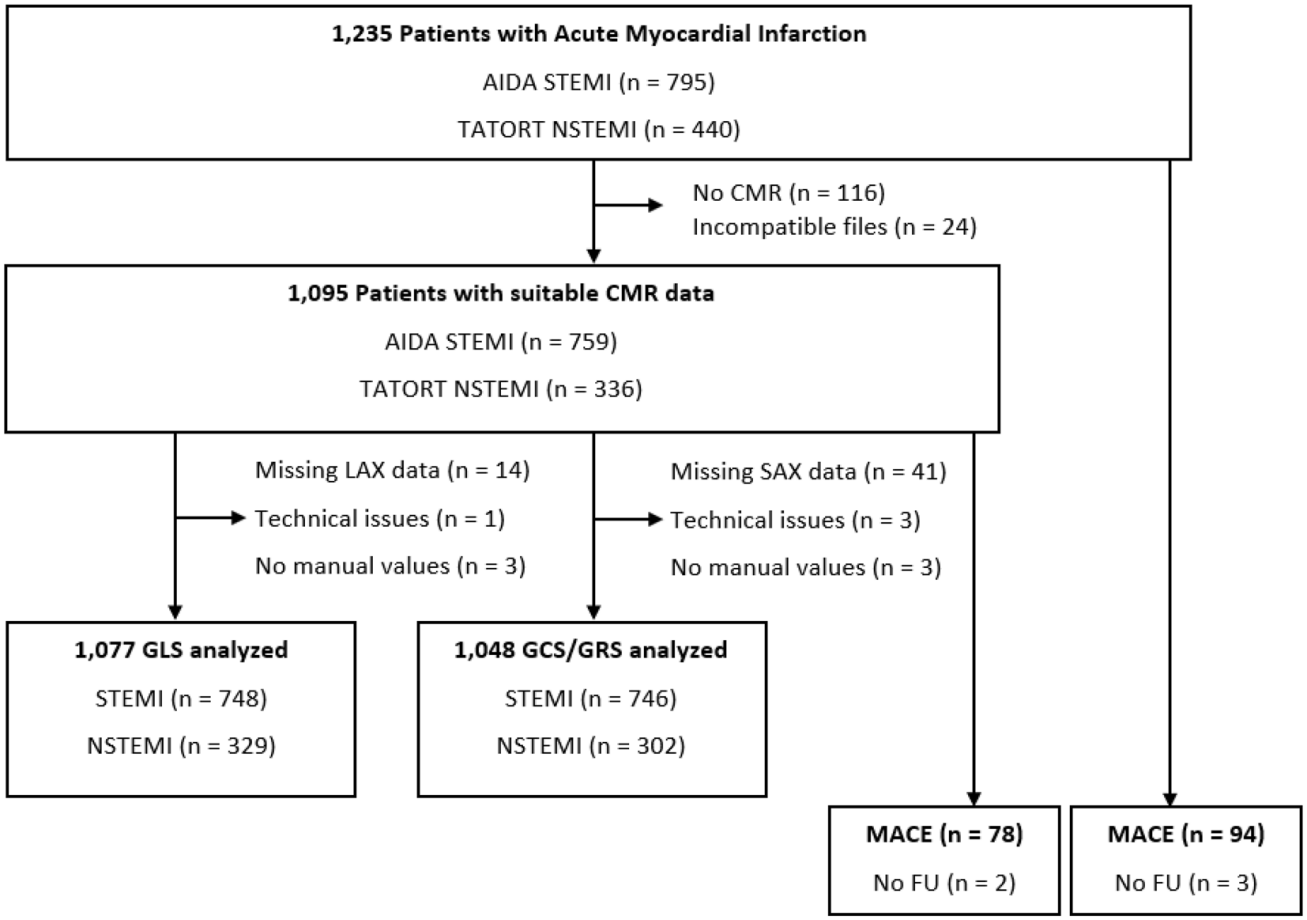
Flow chart of study data. AIDA STEMI, Abciximab i.v. versus i.c. in ST-elevation Myocardial Infarction; CMR, cardiac magnetic resonance; FU, follow-up; MACE, major adverse cardiac events; NSTEMI, non-ST-segment–elevation myocardial infarction; STEMI, ST-segment–elevation myocardial infarction; and TATORT NSTEMI, Thrombus Aspiration in Thrombus Containing Culprit Lesions in Non-ST-Elevation Myocardial Infarction.

The flow-chart for the CMR substudy is shown in Fig. 1. In total, 1235 patients were referred for CMR imaging following AMI (STEMI, n = 795 and NSTEMI, n = 440). Participants with typical CMR contraindications^15^ and patients with missing data or data of insufficient quality for manual postprocessing were excluded. This resulted in a dataset of 1095 patients (STEMI, n = 759 and NSTEMI, n = 336) or rather n = 1077 long axis (LAX) cine sequences for GLS as well as n = 1048 short axis (SAX) datasets for GCS and GRS assessment. The clinical endpoint of the study was defined as the occurrence of major adverse cardiac events (MACE) within 12 months. These included, in order of study priority, all-cause mortality, reoccurrence of an AMI and congestive heart failure. If more than one MACE occurred, then only one was included based on the priority order.

### CMR imaging

CMR cine sequences were acquired using clinical 1.5 or 3 Tesla scanners. The standardised imaging protocol included steady-state free precession imaging (SSFP, repetition time, 3.2 ms; echo time, 1.2 ms; flip angle, 60°; slice thickness, 8 mm) for the acquisition of 2 and 4 chamber views (CV) LAX as well as SAX cine sequences^16^.

#### Manual strain analysis

Manual strain analyses were performed by an experienced investigator using feature-tracking post-processing software (2D CPA MR, Cardiac Performance Analysis, Version 1.1.2, TomTec Imaging Systems, Unterschleissheim, Germany). Manual analyses comprised global longitudinal strain (GLS) derived from 2 and 4 CV long axis cine sequences as well as global circumferential and radial strain (GCS/GRS) averaged from basal, midventricular, and apical locations of a short axis (SAX) cine sequence. Slice selection was performed based on the following criteria: The apical slice was required to have the blood pool present during the entire cardiac cycle. The basal slice must not include the LV outflow tract in any frame. The midventricular slice was chosen in between the apical and basal slice in the presence of the papillary muscles. GLS and GCS were obtained endocardially whilst GRS values were analysed for the myocardium after also placing an epicardial contour. Manually analysed strain values were used as the reference standard to evaluate reproducibility of automated AI derived strain values.

#### Automated strain analysis

**Figure 2:**
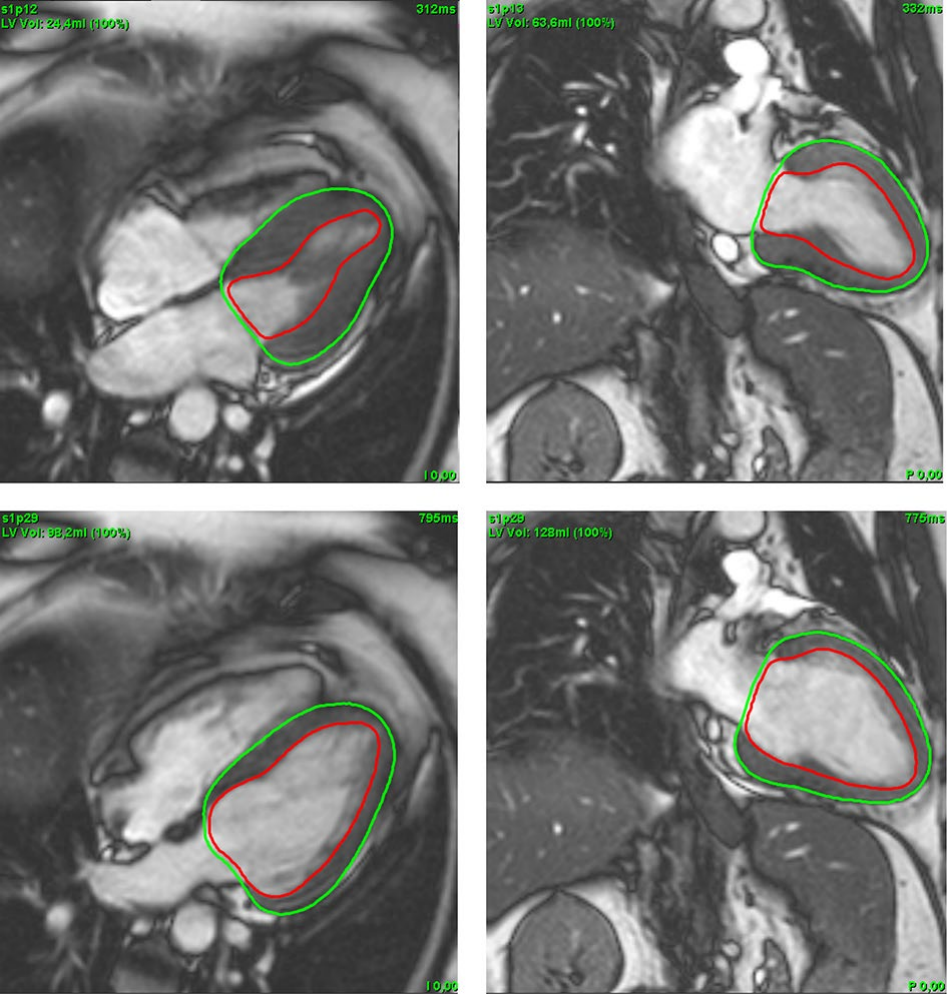
Cardioavascular magnetic resonance LAX images with automated contouring at end systole (top) and end diastole (bottom); 4CV (left) and 2CV (right). 2CV, 2 chamber view; 4CV, 4 chamber view; LAX, long axis.

**Figure 3:**
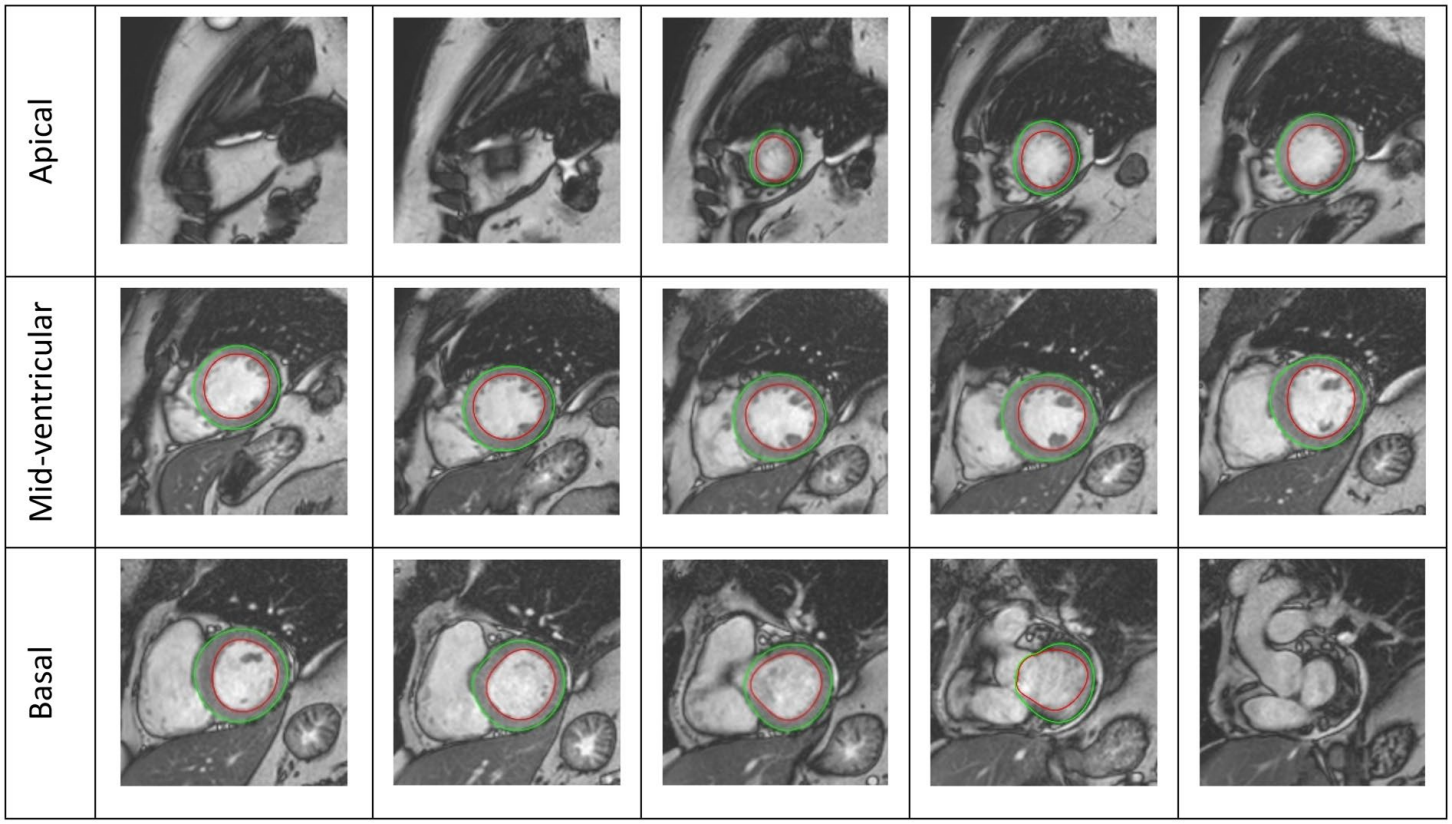
SAX image slices with automated contouring starting from the apical view and ending with the outflow tract of the LV. LV, left ventricle; SAX, short axis.

Automated analyses were performed using commercially available dedicated post-processing software (suite-HEART, v4.0.6; Neosoft, Pewaukee, WI, USA). Prior to the fully automated strain assessment, epi- and endocardial borders of the LV were traced by the algorithm for LAX Fig. 2 and SAX Fig. 3 cine sequences. No user interaction took place for defining the extent of the LV from the most apical to the most basal slice as well as the contouring process. Whilst for GLS, similar to its manual counterpart, one global endocardial strain value for each 2 and 4 CV is reported by the automated software, GCS and GRS are reported for all slices covering the entire LV. Reproducibility of GLS was tested for the average strain of both the 2 and 4 CV. As for GCS and GRS, two approaches were chosen acknowledging the different approaches of manual (three slices) and automated (all slices) analyses. First, to meet the workflow of the manual analyses, the apical, midventricular and basal slice in automated analyses were manually defined by the observer (supplementary Figure S1), an average strain value was calculated for these three slices only. Second, the average for all slices as chosen by the automated software was taken into consideration for comparison to manual assessments.

### Statistical analysis

Statistical analyses were performed using SPSS Statistics, v28, (IBM, Armonk, New York, USA) and MedCalc Version 20.011 (MedCalc Software bvba, Ostend, Belgium). Categorical variables are reported as absolute numbers with corresponding percentages and were compared using the Chi-Squared test. Continuous variables are reported as median with interquartile range and were compared using the Mann–Whitney-U test if independent or the Wilcoxon signed-rank test if dependent, respectively. Agreement of manual vs automated strain values are reported using the intraclass correlation coefficient (ICC) in a model of absolute agreement. An excellent agreement is considered at values greater than 0.74, values from 0.60 to 0.74 good, from 0.40 to 0.59 fair and anything less than 0.40 would have poor agreement^17,18^. Non-parametric correlation was assessed using the Spearman’s rank correlation coefficient. The coefficient of variation (CoV) was calculated by taking the standard deviation of the difference and dividing it by the mean^19^. Bland–Altman plots were used to visualise the difference between the data sets and their outliers^20^, the bias was calculated as the difference between the means of each method. Furthermore, 95% limits of agreement (LOA) were calculated as the mean difference ± 1.96 SD of the mean difference. Univariate Cox regression analyses were used to calculate hazard ratio (HR) and are reported with corresponding confidence intervals (CI) of 95%. Multivariate analyses included univariate significant variables, excluding manual strain values due to high correlation between manual and automated strain values. Kaplan–Meier curves were applied for clinical end point assessment with the cut-off point defined as the median of each variable. Diagnostic accuracy is shown by the area under the curve (AUC) calculated from receiver operating characteristics (ROC). Manual and automated AUC were compared using the method proposed by DeLong et al.^21^. All *p*-values provided are two-sided and were considered statistically significant below 0.05.

## Results

### Study population

Baseline characteristics according to type of AMI as well as occurrence of MACE are reported in Table 1. Baseline characteristics for STEMI and NSTEMI patients are shown in the supplementary Table S1. Patients underwent CMR imaging in median 3 days following AMI. During the 12 months follow-up period n = 78 patients experienced MACE. In addition to elevated age (*p* < 0.001), cardiovascular risk factors such as hypertension and diabetes mellitus were significantly more common in patients with MACE (*p* = 0.014 and *p* = 0.008 respectively). The Killip class on admission was significantly higher in patients with MACE (*p* < 0.001), so was the number of diseased vessels (*p* = 0.010). Both the thrombolysis in myocardial infarction (TIMI) flow grade before and after PCI were not significantly related to the increase of MACE occurrence (*p* ≥ 0.177).

**Table 1:** Baseline characteristics.

Variables	All patients (n = 1095)	MACE (n = 78)	No MACE (n = 1015)	*p* value
Age (years)	64 (53–72)	72 (61–77.25)	63 (52–72)	< 0.001*
Sex (male)	820/1095 (74.9)	52/78 (66.7)	767/1015 (75.6)	0.081
**Cardiovascular risks factors**				
Active smoking	443/1015 (43.6)	22/71 (31.0)	420/1015 (41.4)	0.026*
Hypertension	778/1093 (71.2)	65/78 (83.3)	711/1013 (70.2)	0.014*
Hyperlipoproteinemia	414/1087 (38.1)	27/78 (34.6)	386/1007 (38.3)	0.515
Diabetes mellitus	259/1092 (23.7)	28/78 (35.9)	230/1012 (22.7)	0.008*
Body mass index (kg/m2)	27.45 (24.95–30.35)	27.34 (25.27–31.04)	27.45 (24.91–30.2)	0.685
Previous Myocardial infarction	73/1093 (6.7)	5/78 (6.4)	67/1013 (6.6)	0.944
ST-segment elevation	759/1095 (69.31)	52/78 (66.7)	707/1015 (69.7)	0.581
Time symptoms to balloon, *min	180 (110–316)	192 (115.75–372.75)	180 (110–310)	0.344
Door-to-balloon time, *min	30 (22–42)	27.5 (22.5–39.5)	30 (22–42)	0.429
**Killip class on admission**				< 0.001*
1	967/1095 (88.3)	50/78 (64.1)	915/1015 (90.1)	
2	88/1095 (8)	18/78 (23.1)	70/1015 (6.9)	
3	23/1095 (2.1)	5/78 (6.4)	18/1015 (1.8)	
4	17/1095 (1.6)	5/78 (6.4)	12/1015 (1.2)	
**Diseased vessels**				0.010*
1	546/1095 (49.9)	28/78 (35.9)	517/1015 (50.9)	
2	327/1095 (29.9)	25/78 (32.1)	302/1015 (29.8)	
3	222/1095 (20.3)	25/78 (32.1)	196/1015 (19.3)	
**Affected artery**				0.134
Left anterior descending	450/1095 (41.1)	41/78 (52.6)	409/1015 (40.3)	
Left circumflex	227/1095 (20.7)	15/78 (19.2)	210/1015 (20.7)	
Left main	6/1095 (0.5)	1/78 (1.3)	5/1015 (0.5)	
Right coronary artery	405/1095 (37)	20/78 (25.6)	385/1015 (37.9)	
Bypass graft	7/1095 (0.6)	1/78 (1.3)	6/1015 (0.6)	
**TIMI flow grade before PCI**				0.604
0	551/1095 (50.3)	44/78 (56.4)	506/1015 (49.9)	
1	126/1095 (11.5)	6/78 (7.7)	120/1015 (11.8)	
2	218/1095 (19.9)	14/78 (17.9)	203/1015 (20)	
3	200/1095 (18.3)	14/78 (17.9)	186/1015 (18.3)	
Stent implanted	1068/1095 (97.5)	76/78 (97.4)	990/1015 (97.5)	0.661
**TIMI flow grade after PCI**				0.177
0	21/1095 (1.9)	1/78 (1.3)	20/1015 (2)	
1	23/1095 (2.1)	4/78 (5.1)	19/1015 (1.9)	
2	82/1095 (7.5)	8/78 (10.3)	74/1015 (7.3)	
3	969/1095 (88.5)	65/78 (83.3)	902/1015 (88.9)	
Time to MRI, days	3 (2–4)	3 (2–4)	3 (2–4)	0.022*

Patient data are represented either by n/N (%) or median (interquartile range). *P* values compare the variables with the occurrence of MACE. Two patients were lost to follow up (MACE).

PCI, Percutaneous coronary intervention; MACE, major adverse cardiac event; TIMI, Thrombolysis in myocardial infarction.

*Indicates statistical significance. Mann–Whitney U test was performed for continuous variables and Chi-square test was performed for categorical variables.

### Agreement of manual and automated strain analyses

**Figure 4:**
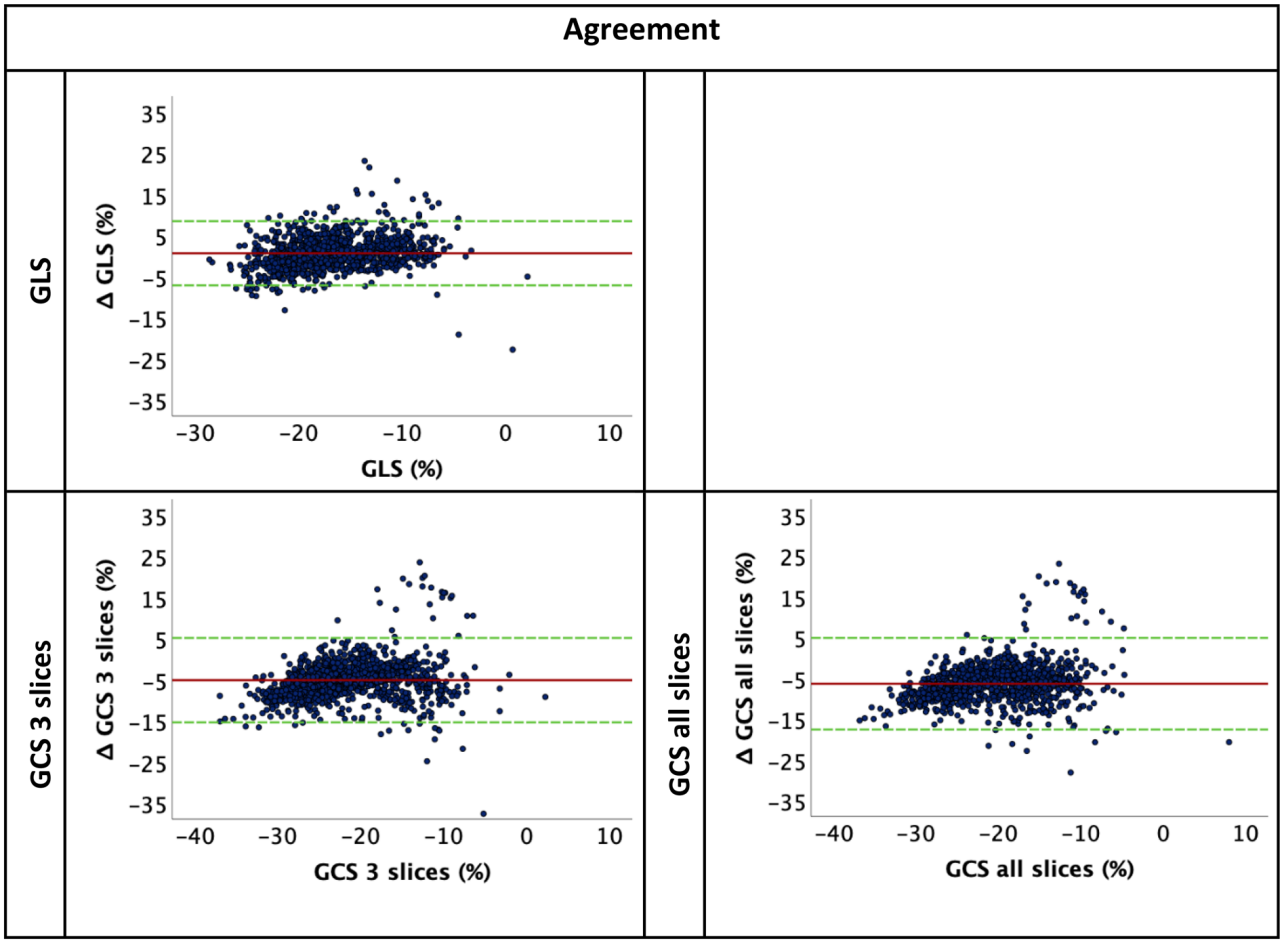
Bland-Altmann plots for agreement of manual and automated strain; GLS, GCS 3 slices and GCS all slices. Agreement between manual and automated strain values represented by Bland-Altmann plot, y axis represents the difference (manual-automated) and x axis is the mean of manual and automated values. GCS, global circumferential strain; GLS, global longitudinal strain.

Automated strain values were higher compared to manually derived GLS (− 17.55% vs − 16.37%, *p* < 0.001) as well as GRS (3 slices: 69.66%/all slices: 70.51% vs 20.45%, *p* < 0.001 for both). In contrast, GCS automated strain values were lower compared to manual analyses (3 slices: − 19.51%/all slices: − 18.48% vs. − 23.83%, *p* < 0.001 for both) Table 2. Agreement between automated and manual cardiac strain values is reported in Table 3. GLS values had the best and excellent agreement (ICC: 0.81, CoV: 24.10%). GCS had a good agreement (3 slices: ICC: 0.68, CoV: 24.87%; all slices: ICC 0.60, CoV: 28.01%). A poor agreement was found for all GRS parameters, (3 slices: ICC: 0.09, CoV: 46.20%; all slices: ICC: 0.09, CoV: 46.20%). The corresponding Bland–Altmann plots are shown in Fig. 4, GRS plots are shown in the supplementary Figure S2.

**Table 2:** Strain measurements for manual and automated strain; GLS, GCS and GCS all slices, GRS and GRS all slices.

	Automated	Manual	*p* value
GLS (%)	− 17.55 (− 20.10 to − 13.60)	− 16.37 (− 20.05 to − 12.30)	< 0.001
GCS 3 slices (%)	− 19.51 (− 22.83 to − 15.13)	− 23.83 (− 28.63 to − 19.06)	< 0.001
GCS all slices (%)	− 18.48 (− 21.79 to − 13.28)		< 0.001
GRS 3 slices (%)	69.66 (54.38 to 88.44)	20.45 (15.58 to 25.90)	< 0.001
GRS all slices (%)	70.51 (55.70 to 85.00)		< 0.001

Strain measurements are continuous variables represented by median and interquartile range. Wilcoxon signed rank test was used to calculate the *p* value for manual values and their respective automated values. Automated GCS and GRS all slices were compared to the manual method of obtaining the average of 3 slices. GCS, global circumferential strain; GLS, global longitudinal strain; GRS, global radial strain.

**Table 3:** Agreement between manual and automated strain analyses; GLS, GCS 3 slices, GCS all slices, GRS 3 slices and GRS all slices.

Parameter	Bias	95% LOA	ICC (95% CI)	Correlation (ρ) (95% CI)	CoV (%)
GLS	0.69	− 7.11 to 8.49	0.81 (0.78–0.83)	0.72 (0.69–0.75)	24.10
GCS 3 slices	− 5.1	− 15.36 to 5.18	0.68 (0.07–0.85)	0.78 (0.75–0.80)	24.87
GCS all slices	− 6.32	− 17.54 to 4.92	0.60 (− 0.08–0.81)	0.77 (0.75–0.79)	28.01
GRS 3 slices	− 51	− 92.97 to − 9.00	0.09 (− 0.08–0.30)	0.48 (0.44–0.53)	46.20
GRS all slices	− 50.26	− 86.88 to − 13.61	0.09 (− 0.08–0.30)	0.49 (0.44–0.54)	40.62

Automated GCS and GRS all slices were compared to the manual method of obtaining the average of 3 slices. Correlation is represented by Spearman’s ρ.

CoV indicates coefficient of variation; GCS, global circumferential strain; GLS, global longitudinal strain; GRS, global radial strain; ICC, intraclass correlation coefficient; LOA limits of agreement.

GLS, GCS 3 slices and all slices showed high correlation to manual GLS and GCS (r = 0.72, 0.78 and 0.77 respectively). GRS 3 slices and all slices showed lower correlation to manual GRS (r = 0.48 and 0.49 respectively).

### Prognostic value of automated strain

In univariate cox regression, baseline characteristics such as age (*p* < 0.001), hypertension (*p* = 0.016) and diabetes mellitus (*p* = 0.009) emerged statistically significantly associated to an increased risk of MACE. Other clinical factors such Killip class on admission (*p* < 0.001) and number of diseased vessels (*p* = 0.003) were also significantly associated with MACE occurrence Table 4. All three functional parameters GLS/GCS/GRS were significantly associated with MACE occurrence in univariate Cox regression analysis (*p* < 0.001) with GLS showing the highest HR of 1.12, 95% CI 1.08–1.16) Table 4. Univariate strain analyses based on all slices showed similar results and are shown in supplementary Table S2. Multivariate analyses included univariate significant baseline parameters as well as automated strain values for GLS, GCS and GRS considering the 3 slices method Table 4. The all slices method showed similar results and is shown in the supplementary Table S3, multivariate analyses calculated for automated GLS/GCS/GRS separately are shown in supplementary Table S4. Amongst automated derived functional parameters GLS emerged as the only independent predictor of MACE occurrence (HR = 1.12, 95% CI 1.08–1.16, *p* < 0.001) in multivariate analyses.

**Table 4:** Univariate and multivariate Cox regression analysis. Multivariate analysis including automated GLS, GCS 3 slices and GRS 3 slices.

Variables	Univariate HR (95% CI)	*p* value	Multivariate HR (95% CI)	*p* value
Age	1.04 (1.02–1.06)	< 0.001	1.03 (1.00–1.05)	0.012
Sex (male)	1.51 (0.94–2.43)	0.083		
Active smoking	0.57 (0.34–0.95)	0.031		
Hypertension	2.07 (1.14–3.76)	0.016		
Hyperlipoproteinemia	0.86 (0.54–1.37)	0.533		
Diabetes mellitus	1.85 (1.16–2.94)	0.009		
Body mass index, kg/m2	1.01 (0.96–1.06)	0.564		
Killip class on admission	2.08 (1.66–2.61)	< 0.001	1.55 (1.14–2.09)	0.004
No. of diseased vessels	1.49 (1.14–1.96)	0.003	1.35 (1.02–1.83)	0.048
Manual GLS	1.13 (1.09–1.18)	< 0.001		
Automated GLS	1.12 (1.08–1.16)	< 0.001	1.10 (1.04–1.15)	< .001
Manual GCS	1.08 (1.05–1.11)	< 0.001		
Automated GCS 3 slices	1.07 (1.05–1.10)	< 0.001		
Manual GRS	0.93 (0.90–0.97)	< 0.001		
Automated GRS 3 slices	0.98 (0.97–0.99)	< 0.001		

Univariate and multivariate analysis represented by HR and 95% CI. Univariate significant parameters (*p* < 0.05) were included in multivariate analysis. Considering high correlation of automated and manual analyses, multivariate analyses were based on automated strain analyses only. GCS, global circumferential strain; GLS, global longitudinal strain; GRS, global radial strain; HR, hazard ratio; CI, confidence interval. Results of multivariable modelling including manual GLS but not automated strain is reported elsewhere^7^.

Another multivariate model was built to compare manual LVEF and GLS to automated analyses (supplementary Table S5). In addition to patients characteristics angiographic data and CMR derived tissue characterisation, either manual LVEF and GLS or automatically derived LVEF and GLS were included to the multivariate analyses. Both parameters performed equally with manual or automated GLS being an independent predictor for MACE (manual GLS HR 1.12 95% CI 1.05–1.18, *p* < 0.001 and automated GLS HR 1.15 95% CI 1.06–1.24, *p* = 0.001).

**Figure 5:**
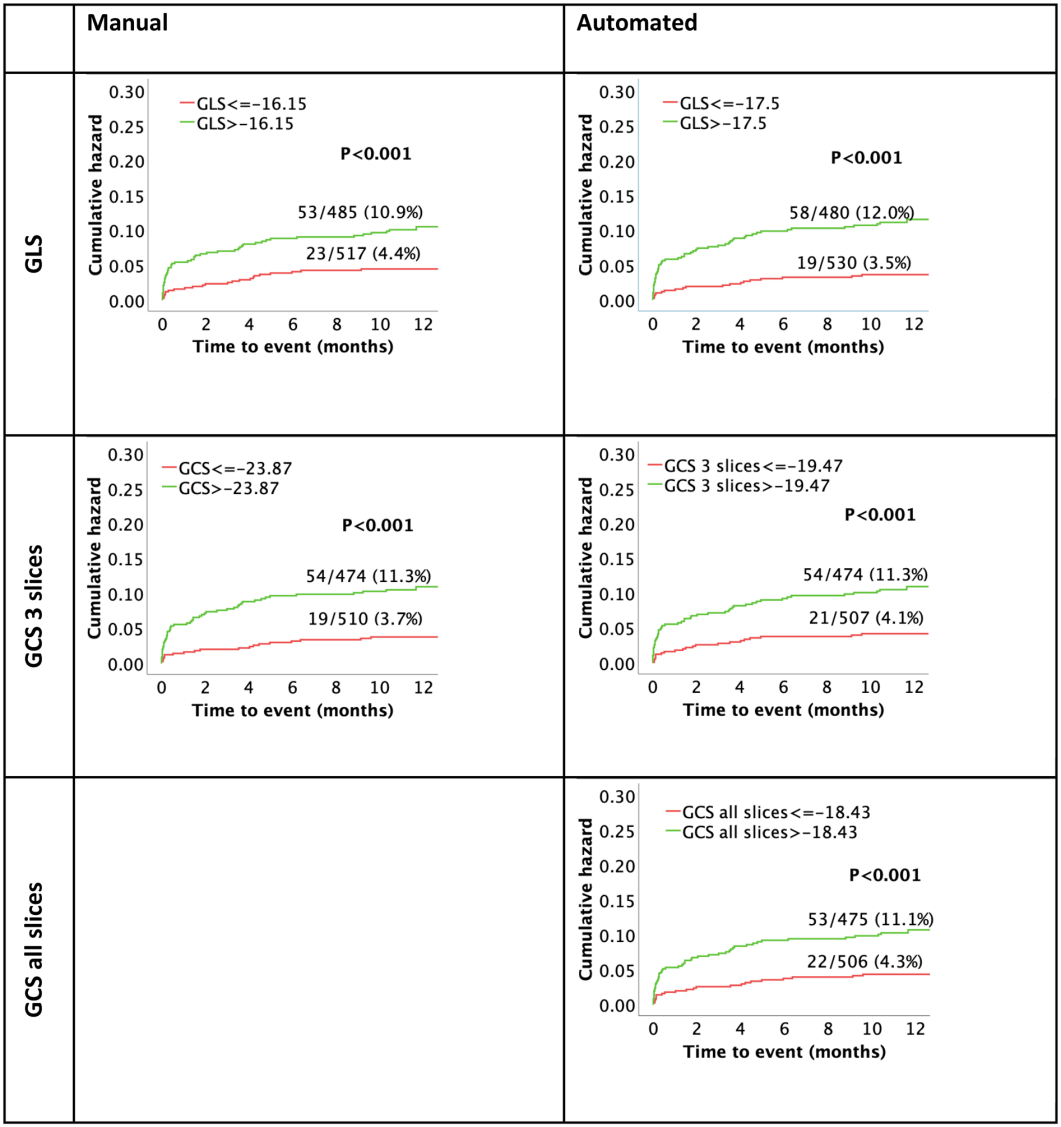
Kaplan–Meier curves assessing survival for manual and automated GLS and GCS. All values dichotomized by their respective medians, time to event represents time to MACE. GCS, global circumferential strain; GLS, global longitudinal strain; MACE, major adverse cardiac events.

Dichotomization at the median of respective strain values was performed to assess risk stratification using Kaplan–Meier curves Fig. 5. GRS curves are shown in the supplementary Figure S3. Both manual and automated analyses of GLS and GCS were significantly associated with MACE (*p* < 0.001 for all). As appreciated from AUC statistics, automated analyses were non-inferior for risk prediction compared to the reference standard of manual assessment: GLS (0.691 vs 0.693, *p* = 0.801), GCS (3 slices: 0.668/all slices: 0.646 vs 0.686, *p* = 0.425/0.055) and GRS (3 slices 0.630/all slices: 0.640 vs 0.642, *p* = 0.537/0.827) Table 5. ROC curves are included in supplementary Figure S4.

**Table 5:** AUC in ROC analysis for manual and automated strain values; GLS, GCS 3 slices and GCS all slices, GRS 3 slices and GRS all slices.

Parameter	Manual	Automated	*p* value
GLS	0.693	0.691	0.801
GCS 3 slices	0.686	0.668	0.425
GCS all slices		0.646	0.055
GRS 3 slices	0.642	0.630	0.537
GRS all slices		0.640	0.827

The AUC was extracted from the ROC graph for manual and automated strain analysis. *P* was calculated with using the DeLong et al. approach^21^. Automated GCS and GRS all slices were compared to the manual method of obtaining the average of 3 slices. AUC, area under the curve; GCS, global circumferential strain; GLS, global longitudinal strain; GRS, global radial strain. ROC, receiver operating characteristic.

## Discussion

The present study investigated the clinical feasibility of novel AI-derived deformation imaging in a large population of prospectively recruited patients who underwent CMR imaging following AMI. Similar to previously published results on manual analyses^7^, GLS emerged as the best and only independent predictor for MACE amongst functional parameters. Second, GLS showed the best and excellent reproducibility compared to its manually assessed counterpart. Last, fully automated AI derived strains may help to implement deformation imaging within clinical routine by cutting down on post-processing times and costs. However, to date, fully-automated results will still need to be confirmed by a clinician who takes responsibility for the management of the patient.

Deformation imaging has shown improved risk prediction in comparison to volumetric analyses^7^ in both ischemic and non-ischemic heart disease^22,23^. Indeed, previous studies have consistently shown that, amongst deformation imaging parameters, longitudinal strain has the highest power for MACE prediction^7,24,25^. In accordance, the present results demonstrate that automated derived GLS best predicted MACE with similar accuracy as appreciated from ROC analyses compared to the reference standard of manual analyses. Similar results for equally accurate risk prediction comparing automated and manual analyses were found for GCS and GRS, however, automated GLS emerged as an the only independent predictor of MACE amongst automated functional assessments which is in line with results shown for manual assessments^7^.

Strain values have been evaluated using different methods in previous studies^7,26^. Unfortunately, its clinical availability is still limited due to the lack of standardised reference values caused by limited agreements between respective approaches for strain assessment and even limited agreements between different software vendors for a specific strain approach^26^. In the present study, especially longitudinal and circumferential strain values highly correlated with manually derived FT values. This is in line with previously shown high intra- and inter-observer reproducibility for FT GLS and GCS^24^. In contrast, absolute agreements comparing manual to automated strains showed higher variations with GLS being under- and GCS being overestimated by automation. Previous data from non-commercially available deep-learning algorithms have reported higher correlation values of GLS and GCS^27^ whilst a study based on echocardiography has reported similar reproducibility of manual and automated assessments for GLS^28^. Notwithstanding, GLS emerged as the parameter with the highest agreement and an absolute bias of below 1.5%. In contrast, GRS was found to be inflated in automation. This could be due to the difficulty of achieving the value of change of thickness of the radius, considering it is relatively small, which could introduce significant errors. It is generally considered a relatively unreliable measure^29^. In the present setting, the automated software did not directly provide the equivalent to manual strain measurements because the automated software derives strain values for the entire ventricle rather than a basal, midventricular and apical slice in manual analyses. The latter is done in manual analyses only to save time without compromising diagnostic accuracy^10^. In that regard, we tested reproducibility to manual analyses first comparing the exact value given by the automated analyses without any observer interference (all slices) as well as three manually selected slices from the automated analyses matching the same selection criteria chosen for manual assessment. Notwithstanding, when comparing reproducibility between manual and automated analyses based either on average strain values from all the slices or from the three manually selected slices, similar results were found. Besides, this also indicates that manual analyses based on basal, midventricular and apical SAX assessment represent overall myocardial function adequately.

Using AI is progressing in the clinical field, especially regarding cardiovascular medicine^30^. This can be achieved by applying machine learning algorithms, which could improve patient care, is cost effective and could reduce mortality rates. Traditional clinical methods have been compared to AI methods in predicting coronary obstructive disease with AI displaying higher sensitivity^31^. It was also shown that machine learning could aid in risk prediction of patients with suspected coronary disease with the support of computed tomographic angiography parameters as opposed to using these parameters alone^32^.

Usually, volumetric analysis and late gadolinium enhancement are used for prediction of MACE but measuring strain has shown to have promising results in adverse event prediction^7,33^. Strain could be better at adverse event prediction than volumetric analysis (LVEF)^33^ but both should be taken into consideration in the clinical setting, as together they could act as a strong risk prediction tool. Using AI based automation software in determining strain shortens the post-processing period and may be implemented to the clinical routine to save time and costs. Indeed, it can be applied on bSSFP cine sequences while perfusion or LGE imaging within the CMR protocol is still being performed. However, results still need to be confirmed by the operator, considering outlier measurements occurred in the automated analysis with extreme values such as positive GLS or GCS and zero strain values. Additionally, the software might detect false borders and would calculate the strain based on those borders. Unfortunately, advances in AI based automated analyses do not address the issue of inter-vendor comparability as an ongoing issue delaying clinical implementation. Furthermore, methodological differences in strain assessment need to be taken into consideration representing^26^ a further obstacle to overcome for AI-based automated strain assessment. Future approaches in AI based risk evaluation in cardiovascular disease may be based on comprehensive cardiac analyses beyond functional evaluations including quantification of LGE and microvascular obstruction (MVO)^11^. Notwithstanding, in contrast to volumetric and strain analyses, the latter still requires manual interaction to differentiate LGE and MVO in infarcted areas. Consequently, for automated comprehensive cardiac functional analyses and tissue characterisation parallel to image acquisition, further developments are warranted. Such future developments combining myocardial shape and function have recently been described and may even further expand our options for fully AI based quantification of cardiac phenotypes with potentially even better prediction of clinical outcome and management of cardiac therapies^34^.

### Study limitations

The data collected for this study was obtained in multiple centres using different CMR vendors. However, the study protocol was the identical. For CMR image acquisition, patients need to be stable enough to undergo the process. Therefore, there might be a selection bias in the selection of the study cohort. Due to the dynamic formation of necrosis and beginning of cardiac remodelling post-AMI, measuring strain after a longer preceding myocardial infarct could lead to an improved prognostic value, however this is not evaluated in the study. The specifications of the algorithm used for the AI software and the deep learning methods are not disclosed by the manufacturer. Thus, the deep learning models could not be properly detailed. Only 2 and 4 CV were available for GLS assessment, nevertheless the progonostic value of GLS derived from 2/4 CV analyses has been demonstrated for MRI^7^ and echocardiography if for image quality not all 3 views can be obtained^35^.

## Conclusion

AI based automated GLS assessment shows similarly high diagnostic accuracy and excellent agreement compared to the reference standard of manually derived GLS. AI based automated strain assessment of GLS representing the most clinically relevant parameter may thus emerge to cut down on post-processing time and costs. If remaining issues such as low inter-vendor agreements between different software types and the absence of uniform reference values can be adequately addressed this technology may enable widespread adoption of CMR GLS measurements in clinical routine practice.

## Supplementary Information

Supplementary Information.

## Data Availability

Regarding data availability, we confirm that all relevant data are within the paper and all data underlying the findings are fully available without restriction from the corresponding author at the University Medical Centre Goettingen for researchers who meet the criteria for access to confidential data.
