# Supplementary material for: Artificial intelligence fully automated myocardial strain quantification for risk stratification following acute myocardial infarction
Source: Sci Rep. 2022 Jul 18;12:12220. doi: 10.1038/s41598-022-16228-w (PMC9293901; doi:10.1038/s41598-022-16228-w)
Supplement: Supplementary file 1 — Supplementary Information. [file 41598_2022_16228_MOESM1_ESM.docx]

**Supplementary material**

| 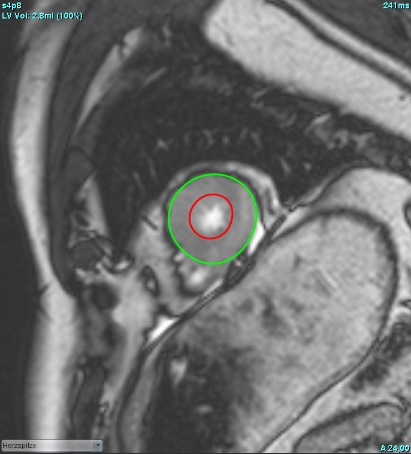 | 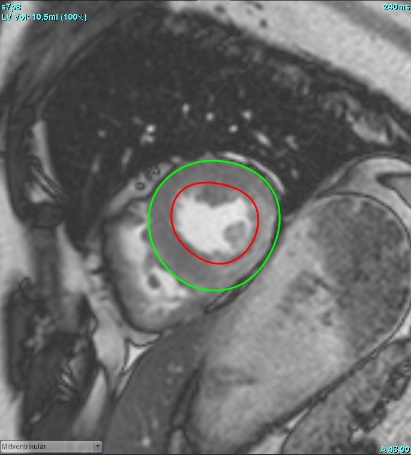 | 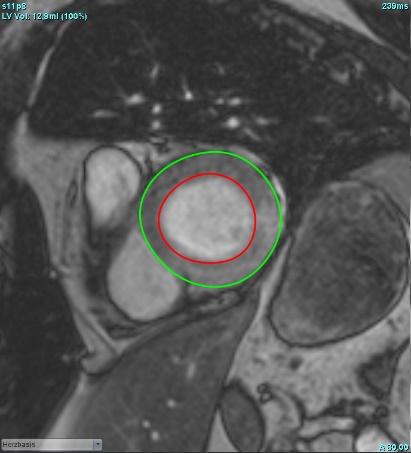 |
| --- | --- | --- |
| 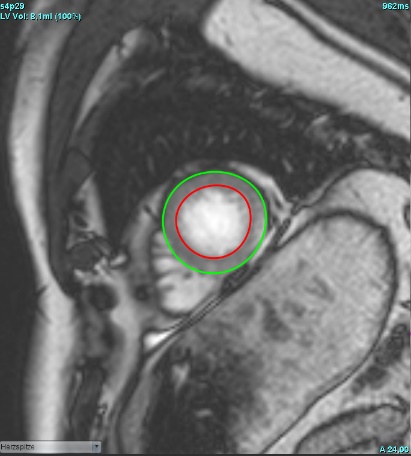 | 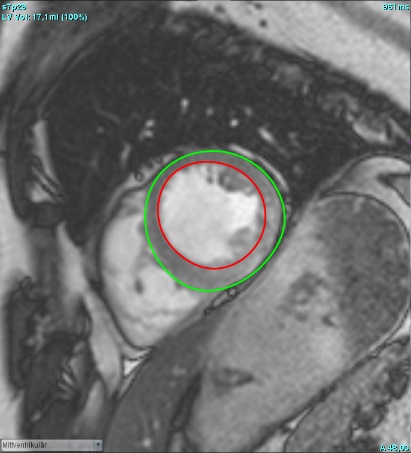 | 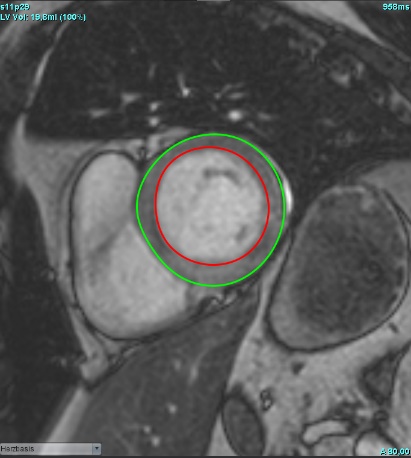 |

**Figure S1. CMR SAX images.**

The Figure shows the three main sections during systole (top) and diastole (bottom); apical (left), mid slice (middle) and basal (right). CMR: cardiac magnetic resonance imaging; SAX: short axis.

| **Agreement** | | | |
| --- | --- | --- | --- |
| **GRS 3 slices** | 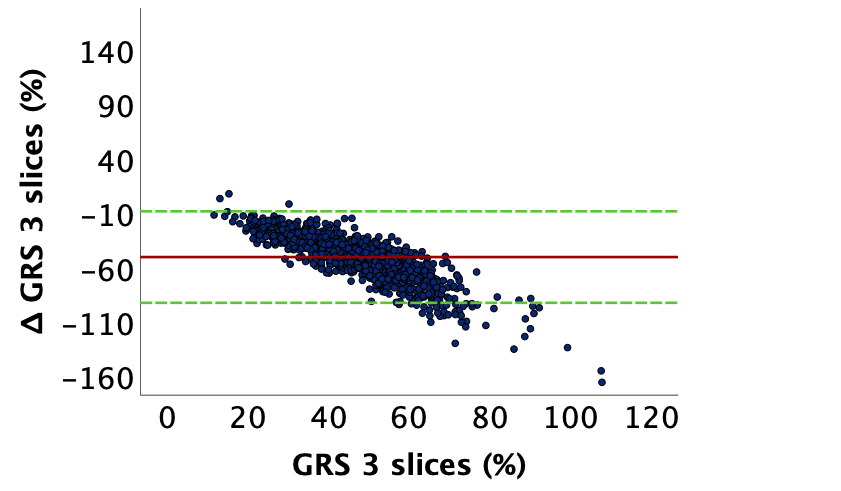 | **GRS all slices** | 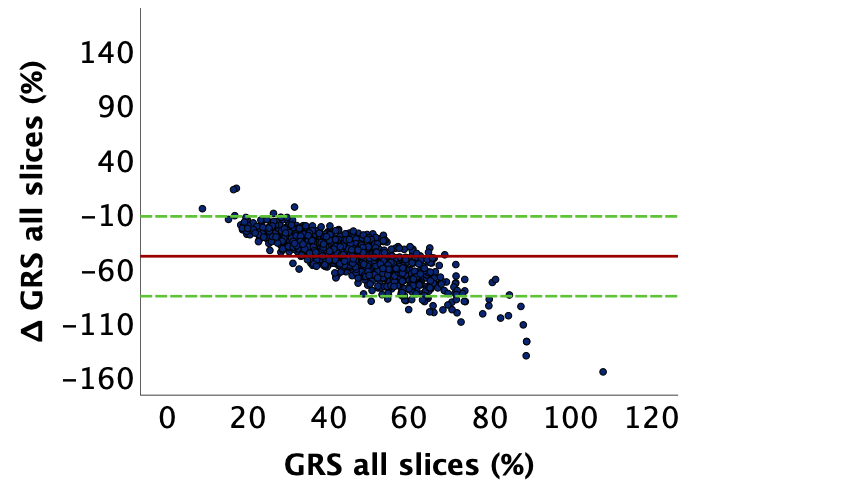 |

**Figure S2. Bland-Altmann plots for agreement of** **manual and automated strain; GRS 3 slices and GRS all slices.**

Agreement between manual and automated strain values represented by Bland-Altmann plot, y axis represents the difference (manual-automated) and x axis is the mean of manual and automated values. GRS, global radial strain.

|  |  | **Manual** | **Automated** |
| --- | --- | --- | --- |
|  | **GRS 3 slices** | 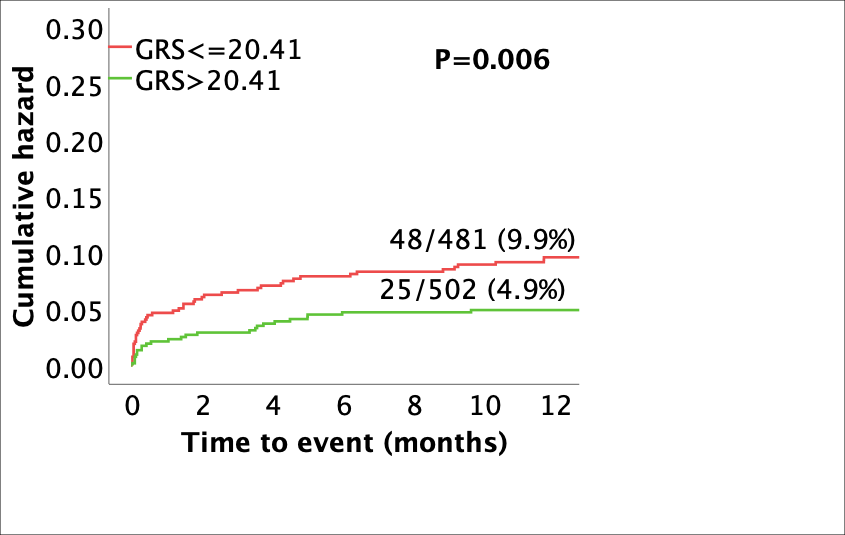 | 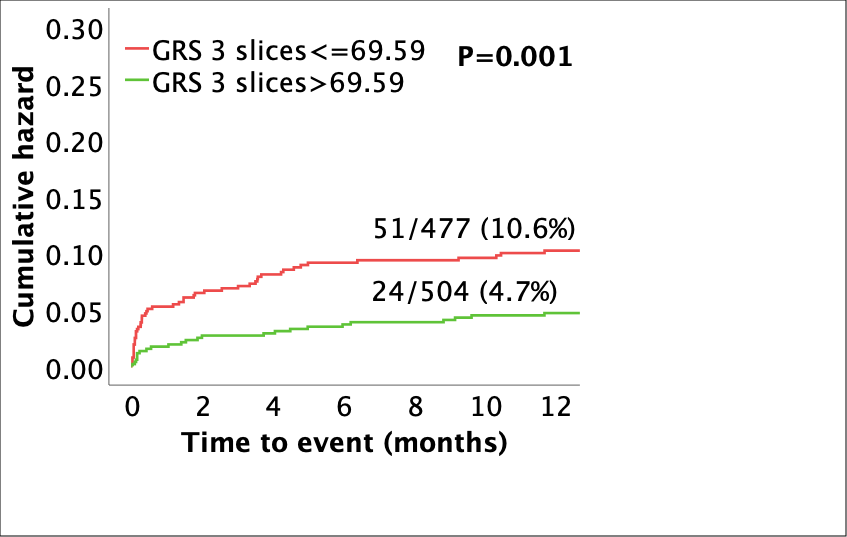 |
|  | **GRS all slices** |  | 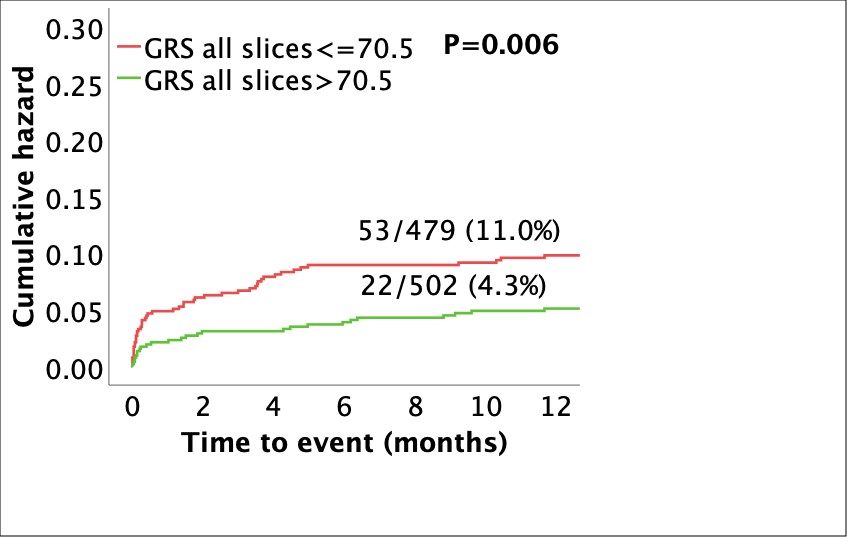 |

**Figure S3. Kaplan-Meier curves assessing survival for manual and automated GRS.**

All values dichotomized by their respective medians, time to event represents time to MACE. GRS, global radial strain; MACE, major adverse cardiac events.

| **Manual** | **Automated** | |
| --- | --- | --- |
| **GLS** | **GLS** |  |
| **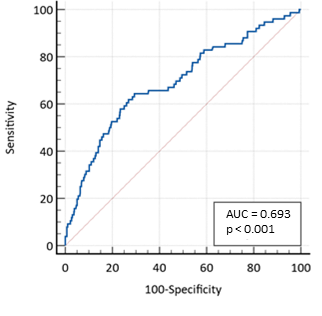** | **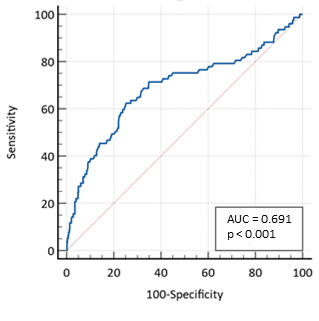** |  |
| **GCS** | **GCS 3 slices** | **GCS all slices** |
| **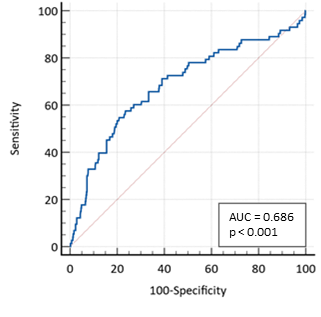** | **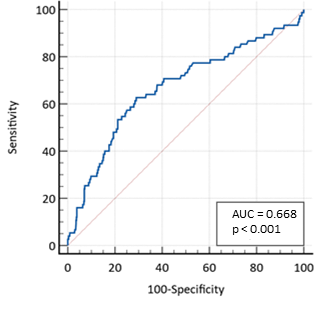** | **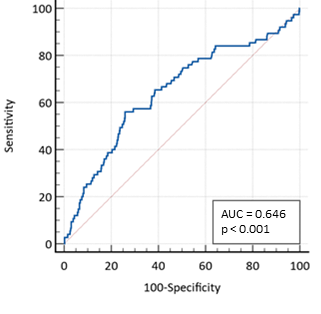** |
| **GRS** | **GRS 3 slices** | **GRS all slices** |
| 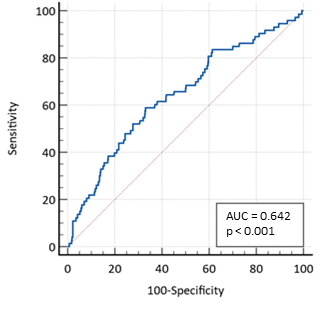 | 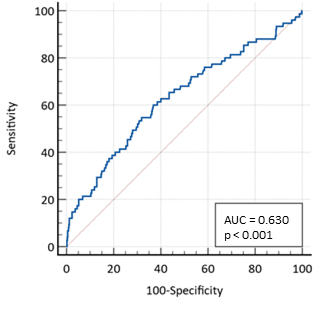 | 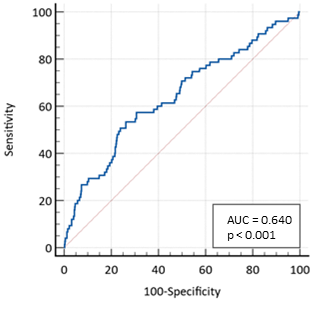 |

**Figure S4. AUC for ROC of GLS, GCS and GRS manual and automated strain values.**

AUC indicates area under the curve; GCS, global circumferential strain; GLS, global longitudinal strain; GRS, global radial strain; ROC, receiver operating characteristics.

**Table S1. Baseline characteristics**

| **Variables** | **STEMI (n=759)** | **NSTEMI (n=336)** |
| --- | --- | --- |
| Age, y | 62 (51-71) | 68 (57-74) |
| Sex (male) | 571/759 (75.2) | 249/336 (74.1) |
| Cardiovascular risks |  |  |
| Active smoking | 323/696 (46.4) | 120/319 (37.6) |
| Hypertension | 517/758 (68.2) | 261/335 (77.9) |
| Hyperlipoproteinemia | 285/752 (37.9) | 129/335 (38.5) |
| Diabetes mellitus | 155/757 (20.5) | 104/335 (31) |
| Body mass index, kg/m2 | 27.34 (24.91-30.24) | 27.72 (24.99-30.48) |
| Previous Myocardial infarction | 42/758 (5.7) | 30/335 (9) |
| ST-segment elevation | 759/759 (100) | 0/759 (0) |
| Time symptoms to balloon, *min | 180 (110-316.5) |  |
| Door-to-balloon time, *min | 30 (22-42) |  |
| Killip class on admission |  |  |
| 1 | 665/759 (87.6) | 302/336 (89.9) |
| 2 | 58/759 (7.6) | 30/336 (8.9) |
| 3 | 20/759 (2.6) | 3/336 (0.9) |
| 4 | 16/759 (2.1) | 1/336 (0.3) |
| Diseased vessels |  |  |
| 1 | 401/759 (52.8) | 145/336 (43.2) |
| 2 | 213/759 (28.1) | 114/336 (33.9) |
| 3 | 145/759 (19.1) | 77/336 (22.9) |
| Affected artery |  |  |
| Left anterior descending | 337/759 (44.4) | 113/336 (33.6) |
| Left circumflex | 87/759 (11.5) | 140/336 (41.7) |
| Left main | 5/759 (0.7) | 1/336 (0.3) |
| Right coronary artery | 328/759 (43.2) | 77/336 (22.9) |
| Bypass graft | 2/759 (0.3) | 5/336 (1.5) |
| TIMI flow grade before PCI |  |  |
| 0 | 422/759 (55.6) | 129/336 (38.4) |
| 1 | 100/759 (13.2) | 26/336 (7.7) |
| 2 | 124/759 (16.3) | 94/336 (28) |
| 3 | 113/759 (14.9) | 87/336 (25.9) |
| Stent implanted | 744/759 (98) | 324/336 (96.4) |
| TIMI flow grade after PCI |  |  |
| 0 | 12/759 (1.6) | 9/336 (2.7) |
| 1 | 19/759 (2.5) | 4/336 (1.2) |
| 2 | 59/759 (7.8) | 23/336 (6.8) |
| 3 | 669/759 (88.1) | 300/336 (89.3) |
| Time to MRI, d | 3 (2-4) | 3 (2-4) |

Patient data are represented either by n/N (%) or median (interquartile range). PCI, Percutaneous coronary intervention; TIMI, Thrombolysis in myocardial infarction.

**Table S2: Univariate analysis including GCS all slices and GRS all slices.**

| **Parameter** | **HR (95% CI)** | **P value** |
| --- | --- | --- |
| Automated GCS all slices | 1.05 (1.03-1.08) | <.001 |
| Automated GRS all slices | 0.97 (0.96-0.98) | <.001 |

Univariate analysis represented by HR and 95% CI. CI, confidence interval; GCS, global circumferential strain; GRS, global radial strain; HR, hazard ratio.

**Table S3: Univariate and multivariate Cox regression analysis. Multivariate analysis including automated GLS, GCS all slices and GRS all slices.**

| **Variables** | **Univariate HR (95% CI)** | **p value** | **Multivariate HR (95% CI)** | **p value** |
| --- | --- | --- | --- | --- |
| Age | 1.04 (1.02-1.06) | <0.001 | 1.03 (1.00-1.05) | 0.010 |
| Sex (male) | 1.51 (0.94-2.43) | 0.083 |  |  |
| Active smoking | 0.57 (0.34-0.95) | 0.031 |  |  |
| Hypertension | 2.07 (1.14-3.76) | 0.016 |  |  |
| Hyperlipoproteinemia | 0.86 (0.54-1.37) | 0.533 |  |  |
| Diabetes mellitus | 1.85 (1.16-2.94) | 0.009 |  |  |
| Body mass index, kg/m2 | 1.01 (0.96-1.06) | 0.564 |  |  |
| Killip class on admission | 2.08 (1.66-2.61) | <.001 | 1.57 (1.16-2.12) | 0.003 |
| No. Of diseased vessels | 1.49 (1.14-1.96) | 0.003 | 1.35 (0.99-1.82) | 0.050 |
| Manual GLS | 1.13 (1.09-1.18) | <0.001 |  |  |
| Automated GLS | 1.12 (1.08-1.16) | <0.001 | 1.10 (1.05-1.15) | <.001 |
| Manual GCS | 1.08 (1.05-1.11) | <0.001 |  |  |
| Automated GCS 3 slices | 1.07 (1.05-1.10) | <0.001 |  |  |
| Manual GRS | 0.93 (0.90-0.97) | <0.001 |  |  |
| Automated GRS 3 slices | 0.98 (0.97-0.99) | <0.001 |  |  |

Univariate and multivariate analyses represented by HR and 95% CI. Univariate significant parameters (p<0.05) were included in multivariate analysis. Considering high correlation of automated and manual analyses, multivariate analyses were based on automated strain analyses only. CI, confidence interval; GCS, global circumferential strain; GLS, global longitudinal strain; GRS, global radial strain; HR, hazard ratio.

**Table S4: Multivariate analysis for GLS, GCS and GRS individually with significant baseline characteristics from univariate analysis.**

| **Variables** | **Multivariate HR (95% CI), GLS** |  | **Multivariate HR (95% CI), GCS 3 slices** |  | **Multivariate HR (95% CI), GRS 3 slices** |  |
| --- | --- | --- | --- | --- | --- | --- |
| Age | 1.03 (1.01-1.06) | 0.003 | 1.02 (1.00-1.05) | 0.029 | 1.02 (1.00-1.05) | 0.017 |
| Sex (male) |  |  |  |  |  |  |
| Smoking |  |  |  |  |  |  |
| Hypertension |  |  |  |  |  |  |
| Diabetes mellitus |  |  |  |  |  |  |
| Killip class on admission | 1.69 (1.28-2.25) | <.001 | 1.57 (1.18-2.08) | 0.002 | 1.68 (1.27-2.22) | <.001 |
| No. Of diseased vessels |  |  | 1.40 (1.04-1.90) | 0.027 | 1.37 (1.02-1.86) | 0.035 |
| Automated GLS | 1.10 (1.06-1.15) | <.001 |  |  |  |  |
| Automated GCS 3 slices |  |  | 1.06 (1.02-1.09) | <.001 |  |  |
| Automated GRS 3 slices |  |  |  |  | 0.98 (0.97-0.99 | 0.021 |

Multivariate analysis represented by HR and 95% CI. Univariate significant parameters (p<0.05) were included in multivariate analysis. Considering high correlation of automated and manual analyses, multivariate analyses were based on automated strain analyses only. CI, confidence interval; GCS, global circumferential strain; GLS, global longitudinal strain; GRS, global radial strain; HR, hazard ratio.

**Table S5: Multivariate analysis for manual vs automated LVEF and GLS**

| **Variables** | **Manual**  **Multivariate HR (95% CI)** | **p value** | **Automated**  **Multivariate HR (95% CI)** | **p value** |
| --- | --- | --- | --- | --- |
| Age | 1.04 (1.01-1.06) | **0.005** | 1.03 (1.01-1.06) | **0.010** |
| Diabetes mellitus | 1.20 (0.66-2.20) | 0.551 | 1.23 (0.68-2.24) | 0.493 |
| Killip class on admission | 1.61 (1.19-2.17) | **0.002** | 1.68 (1.25-2.27) | **0.001** |
| No. of diseased vessels | 1.20 (0.85-1.68) | 0.297 | 1.19 (0.85-1.66) | 0.305 |
| Culprit Lesion | 0.91 (0.61-1.36) | 0.660 | 0.94 (0.64-1.40) | 0.770 |
| Infarct size (%) | 1.00 (0.97-1.03) | 0.770 | 0.99 (0.96-1.02) | 0.527 |
| Microvascular obstruction | 1.02 (0.94-1.11) | 0.706 | 1.02 (0.94-1.11) | 0.616 |
| Manual LVEF | 0.98 (0.96-1.01) | 0.212 |  |  |
| Automated LVEF |  |  | 0.99 (0.96-1.02) | 0.508 |
| Manual GLS | 1.12 (1.05-1.18) | **<0.001** |  |  |
| Automated GLS |  |  | 1.15 (1.06-1.24) | **0.001** |

Multivariate analysis represented by HR and 95% CI for a model including 1. Manually derived left ventricular ejection fraction (LVEF) and global longitudinal strain (GLS) as well as 2. Automatically derived LVEF and GLS.
